# Digital technology and the conservation of nature

**DOI:** 10.1007/s13280-015-0705-1

**Published:** 2015-10-27

**Authors:** Koen Arts, René van der Wal, William M. Adams

**Affiliations:** Forest and Nature Conservation Policy Group, Wageningen University, Droevendaalsesteeg 3, 6700 AA Wageningen, the Netherlands; Centro de Pesquisa do Pantanal, Universidade Federal de Mato Grosso, Cuiabá, CEP: 78.068-360 Brazil; Aberdeen Centre for Environmental Sustainability (ACES), School of Biological Sciences, University of Aberdeen, Aberdeen, AB24 3UU UK; Department of Geography, University of Cambridge, Downing Place, Cambridge, CB2 3EN UK

**Keywords:** Digital conservation, Information and Communication Technology (ICT), The Information Age, Nature conservation, Biodiversity, Innovation

## Abstract

Digital technology is changing nature conservation in increasingly profound ways. We describe this impact and its significance through the concept of ‘digital conservation’, which we found to comprise five pivotal dimensions: data on nature, data on people, data integration and analysis, communication and experience, and participatory governance. Examining digital innovation in nature conservation and addressing how its development, implementation and diffusion may be steered, we warn against hypes, techno-fix thinking, good news narratives and unverified assumptions. We identify a need for rigorous evaluation, more comprehensive consideration of social exclusion, frameworks for regulation and increased multi-sector as well as multi-discipline awareness and cooperation. Along the way, digital technology may best be reconceptualised by conservationists from something that is either good or bad, to a dual-faced force in need of guidance.

## Introduction

The capacity of digital technology to change lives, economies, cultures and societies is universally accepted. Commentators argue that we have entered the ‘Information Age’ (Castells 2010). The internet and associated information and communications technologies (ICTs, e.g. broadband, computers, wireless communication) have created digital networks through which flow large amounts of information. Unlike previous technological revolutions, information is now the central component around which technologies revolve (Castells 2010). This results in new modes of business, communication and governance in many societal domains, including the environmental (Mol 2008).

The digital revolution (involving the use of computers and binary numeric forms of information) is directly relevant to the social practices and organisations concerned with the conservation of nature. Nature conservation is an umbrella term that refers to a plethora of ideas, practices and values, differing for individuals and organisations alike (Adams 2004; Sandbrook et al. 2010). Digital applications have started to gain prominence in nature conservation, in both number and diversity, and are progressively shaping conservation discourses and practices. Digital technology increasingly influences the ways members of the public perceive, think about and engage with nature (Kahn 2011; Verma et al. 2015). The technologies of the Information Age are often greeted with optimism by conservationists because they promise more data, faster processing, better information access and connectivity, new communication routes, exciting visual representations and empowering decision-making support systems. Such optimism may be deceptive in light of the many practical challenges (Joppa 2015; Newey et al. 2015), and the unintended consequences that technology use may bring (Humle et al. 2014; Maffey et al. 2015).

Here we use the term ‘digital conservation’ as shorthand for the broad range of developments at the interface of digital technology and nature conservation (Van der Wal and Arts 2015). We consider the impact and significance of digital technology, understood as the collection of processes and materials related to the innovation, development, implementation and diffusion of digital technology. Our approach draws on Feenberg’s (1999) ‘critical theory’, in which technology is understood as value-laden, and Kranzberg’s (1986, p. 545) ‘First Law of Technology’: “Technology is neither good nor bad; nor is it neutral”. We concur that technology can be understood as a force (cf. Castells 2010) that shows an “ambivalent face, empowering and hindering at the same time” (Lanzara 2009, p. 38), and accept that nature conservation practice, like conservation science, is ‘mission-driven’ (Meine et al. 2006; Mace 2014; Maffey et al. 2015). Therefore, we view it as vital for conservationists to understand how their mission is affected by digital technology.

### Study approach

In this paper, we seek to identify and analyse the application of digital technology in nature conservation. To undertake this analysis, it has been necessary to extend our search beyond peer-reviewed publications and other scholarly works. Formal academic literature is often published following a long delay, thus making it a potentially poor indicator of the current state of affairs. Furthermore, commercial and other non-academic developments, often arising rapidly, are commonly described in grey literature and online sources. Systematic review methodology tends to avoid these in their emphasis on data quality (e.g. Pullin and Stewart 2004). Our approach owes more to horizon scanning exercises, which aim to identify relatively unknown phenomena at the earliest possible stage (Sutherland et al. 2014).

**Fig. 1:**
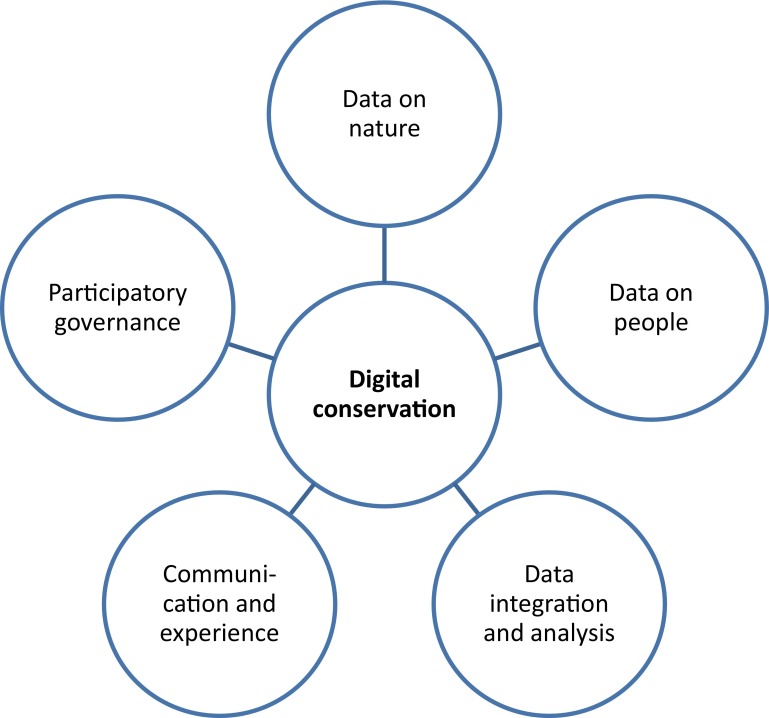
Five key dimensions of digital conservation

We conducted keyword searches with Google Scholar and Web of Science, using search terms related to ‘nature conservation’ and ‘digital technology’.^1^ In addition, we gathered material from participants at the first International Conference on Digital Conservation (21–23 May 2014, Aberdeen, UK) and through Twitter accounts (Amanatidou et al. 2012). Returns were assessed (by title, introduction, abstract, images, and where needed, body text) to derive recurrent themes, which were subsequently grouped (Strauss and Corbin 1998). On the basis of this, we identified five key dimensions which have a substantial impact on nature conservation (Fig. 1). Each dimension, and its most important associated possibilities and problems, is discussed and supported by an illustrative but not exhaustive set of sources (non-peer-reviewed online sources are referred to in footnotes). Although we discuss the identified dimensions separately, their boundaries are fluid. As such, digital conservation follows a pattern identified in other domains with “growing convergence of specific technologies into a highly integrated system, within which old, separate technological trajectories become literally indistinguishable” (Castells 2010, pp. 71–72). In the Discussion, we address the challenge of how to increase benefits associated with digital technology in nature conservation while reducing associated risks.

## Data on nature

### Possibilities

Mass-produced, high-tech sensors and related technology make it possible for there to be more, better, faster and cheaper capture of data on nature (Van Tamelen 2004; Koh and Wich 2012; Will et al. 2014).^2^ These technologies are implemented in various ways, from multi-sensor equipped smart phones carried by humans and satellite tags carried by animals, to camera traps, drones (also called Unmanned Aerial Vehicles or UAVs), deep-sea submarines and space satellites. It has enabled more frequent monitoring of the natural environment, on a larger spatial scale, at a finer resolution in inaccessible or dangerous locations, and has sometimes resulted in (near) real-time sensing (Blumstein et al. 2011; Van der Wal et al. 2015b). Such developments can bring clear benefits to conservation science and management (Pettorelli et al. 2014; August et al. 2015). Many tools also allow automated capture of data: once activated they require no or minimal further human involvement (Waddle et al. 2003; Wagtendonk and De Jeu 2007). Pioneering examples include biomimetic robots such as *iTuna*^3^ or *Cyro*, the latter of which recreates the movement of jellyfish while monitoring marine environments.^4^ A different feature of ‘data on nature’ is that new kinds of data can be generated. Ongoing miniaturisation of technology allows for the tracking of movement of very small animals, right down to insects (Lihoreau et al. 2012).^5^ Integration of different types of sensors (registering e.g. heat, temperature, heart rate)^6^ allows users to make rapid and better informed inferences (Wall et al. 2014). Such integration of different sensors also opens up new ways of turning data into information (Robinson Willmott et al. 2015), for instance through so-called Natural Language Generation, i.e. the automated generation of language based on digital data processing (cf. ‘blogging birds’^7^—Van der Wal et al. 2015b). The omnipresence of smart personal devices has allowed conservation initiatives to encourage both skilled and less-skilled people to contribute to biological recording (Van der Wal et al. 2015a).^8^ Citizen science—i.e. volunteers taking part in a scientific enquiry—is rapidly becoming a paradigm of its own within nature conservation, and is often strongly dependent on digital devices and applications, especially smartphones and related apps (Dickinson et al. 2010; Conrad and Hilchey 2011; Silvertown et al. 2015).^9^ Computer-aided taxonomy and analysis can help relatively unskilled citizens to identify species and process data (Oswald et al. 2007; Walters et al. 2012; Wilson and Flory 2012).^10^ Electronic field guides can replace heavy books and may provide a user-friendly tool for species identification by specialists and non-specialists alike (Stevenson et al. 2003; Farnsworth et al. 2013). Bayesian computer models are used to determine minimum crowd sizes to achieve correct species identification of photographed specimens (Siddharthan et al. 2015). Digital technology can unlock the potential of already collected data, with citizen scientists for example helping with the digitisation of natural history collections (Canhos et al. 2004; Blagoderov et al. 2012). The *Notes from Nature*^11^ project uses crowdsourcing to transcribe biological records. By the beginning of September 2015, 7994 volunteers contributed to 1 160 000 transcribed museum records. Such an example illustrates the potential of these kinds of digital projects to engage a citizen workforce.

### Problems

Sensors and related technologies hold much promise but inherent technical barriers may hinder implementation. For example, widespread use of lower-end camera traps in conservation and wildlife management research proves troublesome due to numerous deployment, operation and data management issues (Meek et al. 2015; Newey et al. 2015). Technology may have negative implications for humans and nature. As Sandbrook (2015) shows, drones could have severe social implications, and actually negatively impact on humans, animals and conservation practices at large if used without appropriate legislative and ethical frameworks (Ditmer et al. 2015; Vas et al. 2015). Another negative impact may materialise through a greater resource and energy consumption and the creation of additional e-waste (Fuchs 2008). Many electronic devices are built with planned obsolescence, and resulting e-waste is largely exported to developing countries where it can create environmental problems (Maffey et al. 2015). The same technologies that contribute to nature conservation can be used for purposes that conflict with conservation aims. For instance, camera traps and drones could be used to enable illegal hunting, and in marine environments technologies such as echo sounders and GPSs facilitate intense fishing and resource depletion (Roberts 2007). Technological development can also be dysfunctional: advances in sensor hardware may outpace those in software (Campbell et al. 2013), and social development processes of apps and websites are often non-inclusive (Teacher et al. 2013). The latter contributes to nature apps not reaching their full potential (‘waiting for the revolution’—Jepson and Ladle 2015). Access to digital devices, technologies and supporting infrastructures (e.g. electricity) and knowledge is globally highly uneven. In a similar way, with regard to digitising natural history specimens, Vollmar et al. (2010, p. 93) found an “uneven digitisation landscape” with “a patchy accumulation of records at varying qualities, and based on different priorities”. Finally, a perverse effect of the automated surveying and identification of species could be ‘de-skilling’ of natural history, as machine-support compensates for a decline in people with taxonomic knowledge.

## Data on people

### Possibilities

With the increased flow of data and information (i.e. interpreted data), a new level of monitoring has become possible, notably through the mining of social networks and through ‘web crawlers’, software scripts that methodologically browse the World Wide Web (cf. Galaz et al. 2009; Stafford et al. 2010; Barve 2014). Search engine data can now not only be used to forecast biological events such as pollen release and mosquito outbreaks, but can also reveal signs of changes in environmental perceptions of internet-using communities (Proulx et al. 2013; Kim et al. 2014; but see also Ficetola 2013). Such techniques extend the field of ‘culturomics’ (the quantitative analysis of cultures—Michel et al. 2010) to nature conservation. These and other approaches make use of the capacity for automated search and analysis of digital data, and allow for a considerably greater geographical reach and sample size of surveys. For instance, the Greendex 2014 survey on environmentally sustainable consumption collected around one thousand responses in each of the 18 focal countries in just over 40 days.^12^ To reach such a high and wide response rate through an analogue survey would have been a costlier and more labour-intensive undertaking (and arguably less likely to have been done). Digital sensing and tracking devices open up the possibility of obtaining continuous, direct data on human activities relevant to nature conservation. Methods such as ‘experience sampling’ employ embedded sensors (e.g. in smartphones) to track human movement. This can inform understanding of the ways in which people use natural environments (e.g. recreation in greenspaces) (Doherty et al. 2014). Data from devices such as camera traps, embedded cameras, GPS tags, drones and satellites can be used to detect or study, for example, illegal wood logging and poaching.^13^ Similar tracking technologies can also be employed to monitor value chains and product lifecycles, and hence provide a foundation for energy and waste reduction (Saar and Thomas 2002) or for a more effective combatting of illegal timber trade.^14^ The rise of the ‘internet-of-things’ (e.g. common household appliances connected to the internet) might promote reduced resource consumption, for example through the remote control of central heating systems, and potentially improve consumer insight into the connections between nature and resource consumption (Miorandi et al. 2012).

### Problems

The potential of digital technology to enable intensified and spatially distributed surveillance, and automated analysis of data, bring significant issues of human impacts and human rights (e.g. Humle et al. 2014; Sandbrook 2015). Mol (2008, p. 116) points out that environmental monitoring has traditionally escaped such criticism because its practices were: (i) too limited in size, capacity and intensity; (ii) more focussed on institutional and market actors than citizens; and (iii) revolved around physical qualities of the environment rather than human actions. However, this is changing. Digital devices are outstripping institutional frameworks for their development, and for the storage and analysis of data collected. There are questions about who should be permitted to deploy such devices (e.g. public or private organisations), where they may be used (on public or private land), and whether people need to be informed about, or consent to, data collection. There are questions about how data may be stored or used, and by whom.^15^ Debates about these issues are current among human rights organisations (e.g. about the implications for civil liberties of surveillance by police or other state organisations) and of great relevance to nature conservation. Scholars note a lack of international regulation, legislation, frameworks and ‘good practice’ guidelines (Finn and Wright 2012; Sandbrook 2015). The use of drones in the battle against poaching may provide a case in point: will tourists be (in)directly affected as a result of wildlife authorities gathering data in a given national park?

## Data integration and analysis

### Possibilities

One result of the rapid development of hardware is the rise of ‘big data’ (Kitchin 2014; Kelling et al. 2015). Data volumes are rapidly increasing (terabytes and petabytes), they are nearer real-time, increasing in scope (capturing entire populations or ecosystems) and finer in resolution. The opportunities offered by big data have been described as “unprecedented (…) for advancing science and informing resource management” (Hampton et al. 2013, p. 156). Big data implies connection of datasets, and a number of initiatives have emerged to promote standardisation and inter-operability of heterogeneous data sources (Jones et al. 2006; Stein 2008). The *Global Biodiversity Information Facility* (*GBIF*) works as a network of nodes (of about 14 000 datasets) and—at the beginning of June 2015—provided a single point of access to more than 500 million records on almost 1.5 million species^16^ (see also the *National Biodiversity Network Gateway*^17^). Similarly, the *Darwin Core* project aims to provide one body of standards for publishing and integrating biodiversity information,^18^ while the *Speciesbank.com*^19^ is a central platform and database for biodiversity market participants. Various aspects related to big data and biodiversity information are central to ‘bioinformatics’ (Soberón and Peterson 2004), a relatively young field with the ultimate goal to develop a commonly shared easy-to-access e-infrastructure, facilitating “the full integration of the biodiversity research community” (Hardisty and Roberts 2013, p. 1). Big data requires new forms of analysis. Aided by fast computer processors and cloud computing, conservation practices may benefit from increasingly sophisticated analyses and modelling for scientific and managerial purposes (Wall et al. 2014; Chapron 2015; Kelling et al. 2015).^20^

### Problems

Big data also presents challenges for nature conservation relating to access, connectivity and analysis (Porter et al. 2012; Kelling et al. 2015). The reluctance of some to use novel technology may be a barrier to uptake, sometimes reinforced by over-complicated user interfaces (Hardisty and Roberts 2013). Other recurrent issues are whether scholars and institutions are willing to share codes and data (Borgman et al. 2007; Peterson et al. 2010), and whether databases are linked up to larger cyberinfrastructures in systems of open access (Borgman et al. 2007; Campbell et al. 2013). There are important questions of who will pay for data collection and maintenance in shared meta-datasets. Associated issues relate to the control of data. There are potential risks for nature conservation when datasets are targeted by hackers (e.g. poachers using web-linked imaging devices to locate rare animals in real-time) or developers (e.g. using conservation datasets to support natural resource extraction planning). Associated with those risks are questions about accountability of those who are controlling such data. Moreover, more data and more analysis do not necessarily aid decision-making. Canhos et al. (2004, p. 1) noted that the budding discipline of bioinformatics was bringing new opportunities and novel approaches to “ecological analysis, predictive modelling, and synthesis and visualisation of biodiversity information”. Yet, a few years later, it was observed that little data sharing had occurred in bioinformatics, and competing platforms had emerged resulting in practices “that have no connection to genuine insight and forward progress” (Peterson et al. 2010, p. 159).

## Communication and experience

### Possibilities

Internet-supported social media have offered lay people and experts new means to self-organise and exchange ideas, experience and footage (e.g. Ashlin and Ladle 2006; Bombaci et al. 2015).^21^ Platforms like *Open Air Laboratories* (*OPAL)*,^22^*eBird*,^23^ the *iNaturalist* App,^24^ the *Atlas of Living Australia*^25^ and *WikiAves*^26^ do not only provide scientists with data, but also allow people to become part of a community through uploading observations of flora and fauna, inspecting sightings by others, and fostering discuss on and learning about the natural world. Digital technology has also impacted on organisation-to-citizen relationships. Conservation organisations and research institutes routinely employ social media, webcam imagery and other tools for all kinds of public engagement-related aims, e.g. to provide information, consult, create interest in specific topics, maintain or win public and political support, or bring people into the conservation fold (Lundmark 2003; Saito et al. 2015; Verma et al. 2015).^27^ Digital technologies can play an important role in knowledge transfer and e-learning, which is encouraging in times when taxonomic skill sets are in decline (Hopkins and Freckleton 2002). They can also play a vital role in motivating and retaining volunteers and others involved in, or engaged with, nature conservation (Van der Wal et al. 2015c). Gaming may contribute to education and behaviour change, fundraising and research (Sandbrook et al. 2014).^28^ Technology-supported games can also encourage children and other players to go into nature more. For example, in the *Wildtime App*^29^ technology is used as a facilitator; children or parents indicate on their mobile phone how much time they have, and on the basis of that a list is returned for enjoyable activities in nearby green space. Virtual representations (e.g. through virtual reality headsets) of nature may be employed for many different or overlapping purposes including recreation, tourism, education and well-being, and could be all the more important in light of growing global urbanisation and disconnect from nature (Turner et al. 2004; Saito et al. 2015).^30^

### Problems

Digital games may prevent gamers from going outside, or have the potential to distract gamers from real-world problems (Sandbrook et al. 2014). It is conceivable that digital representations of the natural world may become a substitute for physical nature: recordings of wild organisms (including individuals now dead or species now extinct), or synthesised quasi-natural environments, might substitute for directly experienced nature. Tests of ‘technological nature windows’ (synthesised natural scenes, for example in offices and hospitals) show that these are (as of yet) not as restorative as actual nature (Kahn 2011). Moreover, with the rise of ICTs, people’s relationship with nature is further mediated through an increasingly complex digital web. White and Wilbert (2009, p. 6) have used the term ‘techno-natures’ in this regard: “knowledges of our world are, within such social natures, ever more technologically mediated, produced, enacted, and contested”. Indeed, nature conservation organisations are not neutral agents in mediating nature through technology (Żmihorski et al. 2013); techno-visual set-ups may stimulate emotional involvement, but turn wildlife into a ‘tele-visual commodity’ (Chambers 2007) “packaged for the purposes of eliciting donations, membership monies, and repeat visits” (Verma et al. 2015). Discussing the example of the internet search engine *Ecosia,*^31^ Büscher (2013) reveals potential negative consequences of social media and other interactive communication modes used by conservation organisations, including the (further) commodification of nature and its conservation. Nature 2.0, as he labels it, represents a new reality in which the political economy of global conservation is increasingly underpinned by digital technology.

## Participatory governance

### Possibilities

A topical dimension of participatory governance is e-governance, i.e. the use of ICTs in state practices. According to some, an evolution towards e-governance 2.0 has been taking place, involving a transformative, participatory model of online interaction between government and citizens (Mathur 2009; Chun et al. 2010; UN 2014). Participatory governance may also involve a wider digital public participation in natural resource management, decision- and policy-making (Arts et al. 2015b). This can be supported with e.g. computer models and GIS mapping exercises,^32^ potentially leading to experiential learning cycles (Haklay 2003; De Kraker et al. 2011; Buytaert et al. 2012). Building on the advantages that cloud computing brings (such as faster processing opportunities and centralised update procedures), Chapron (2015) developed a web-based application for wildlife management driven by a moose population matrix model that quickly provides a hunting quota to users in line with the carrying capacity of selected areas. Digital support systems and e-governance also have a potential for democratisation and social empowerment, particularly with regards to under-represented communities and rural people. Graham et al. (2012) illustrate how a mobile phone-based decision support communication tool can reduce human-elephant conflict, aid conservation more broadly, and empower local people. The *Extreme Citizen Science Group*^33^ has developed participatory mapping technologies which allowed Mbendjele hunter-gatherers in the Congo basin to map activities of commercial poachers (Lewis 2012; Stevens et al. 2013; cf. Rahemtulla et al. 2008 and *Mapping for Rights*^34^).

### Problems

Public authorities and organisations that seek to adopt Governance 2.0 approaches will be faced with numerous barriers to implementation and use. These may relate to, for example, path-dependencies, siloed departments, lack of human and financial resources, conflicting types of knowledge and framing, differing views of staff on the value of digital technology, and bureaucracy (Kamal 2006; Arts et al. 2015a). A problem in wider digital technology discourses is that of digital exclusion. Traditional literatures on the digital divide have focussed on the binary of who uses the Internet and who does not. While large parts of the World indeed remain unconnected to the Internet, more attention has recently been paid to second-order divides including autonomy of Internet use, social support networks, use patterns and skill levels (Hargittai 2002; Warren 2007), but as of yet it is ill-understood how these play out in nature conservation communities. With regard to decision-making support tools, their full potential is often not reached, notably because the intended end-users do not adopt the tool (Tremblaya et al. 2004; De Kraker et al. 2011; McIntosh et al. 2011), a likelihood which is greatly enlarged when a support tool is made *for* a conservation community of users rather than *with* them (Maffey et al. 2013).

## Discussion: Challenges for conservationists

Digital technology is impacting on nature conservation in myriad ways, creating possibilities and problems, as well as winners and losers. Both sets often represent different sides of the same coin. This is not to say that the possibilities and problems of any of the application areas are of equal importance, or in balance. The challenge for conservationists, we argue, is to capitalise on the opportunities while reducing the associated threats.

### Longevity of technology

Nature conservationists increasingly seek to embrace digital technology as a central element of their science, management, communication and other practices, and it is likely they will continue to do so in the future. Many media platforms enforce this enthusiasm by presenting digital technology as a panacea to a suite of conservation problems. Such enthusiasm may be long-lived, i.e. when digital technology becomes a structural component of an organisation’s practices (e.g. an online submission system for a volunteer-based initiative—Arts et al. 2013). But it can also be short-lived: a particular technological application may be employed as a techno-fix that does not address the root cause of a problem (Huesemann and Huessemann 2011), or become a hype, which “usually ends suddenly when the realisation hits that it is not as important as it was thought to be or when the hype has become common practice” (Meijer et al. 2009, p. 3). Nature conservation has always been susceptible to hypes and fads (Redford et al. 2013a), and an emphasis on short-term promises resonates with the mission-driven character of nature conservation (Meine et al. 2006). This could sit at odds with the growing paradigm of evidence-based conservation, in which technology-related promises are not taken for granted, but tested (Sutherland et al. 2004). We argue that nature conservation as a whole would benefit from less emphasis on the short-term promises of digital technology, and more emphasis on their medium- and long-term impacts.

### Bias towards good news narratives and new approach to digital technology

Nature conservation suffers from a tendency to embrace ‘good news narratives’. This bias is not only present in popular media stories, but also in scientific literature at the interface of nature conservation and digital technology, which generally reports little on the challenges, setbacks, backlashes, or failures that many projects face (cf. Arts et al. 2013; Newey et al. 2015). Many digital technology projects seem to die a silent death or not move beyond their pilot phase, for example due to lack of continued project funding, departure of staff, or the academic focus on research questions (Joppa 2015). Sometimes, good news narratives may have less to do with the true possibilities of technology (such as more data or improved efficiency for better nature conservation), and more with an organisation’s desire to use a digital application as a vehicle to impress, to attract attention through novelty, or to make itself look modern and hence to help attract funding. At best, the dominance of stories about the promise of digital technology currently paints a misleading image. At worst it sustains a simplistic and naïve logic that may negatively affect nature conservation in the long run by prematurely closing useful debates, thus impoverishing conservation thinking. We therefore suggest that approaches to digital technology in nature conservation need to change to avoid treating technology as a magic wand to solve conservation problems at a stroke. A more constructive approach to digital technology would be to consider it as a force (Castells 2010). Such a force can perhaps be guided and steered for certain purposes, but not necessarily fully controlled or employed. As of yet, the force of digital conservation is little understood, and a key challenge is to ensure that it feeds less into techno-fix thinking and hypes, and more into long-lasting and carefully implemented applications.

### Political economies and digital exclusion

Questions of who controls, pays for, benefits from, is negatively affected by, or administrates digital technology are questions of political economy that are of outmost importance to nature conservation. In light of conservation’s mixed historical track record with regard to the exercise of power and social impacts (Adams 2004; Brockington et al. 2008), critical examination is required of the application of digital technology, for example regarding the acquisition, storage and use of data. Conceptually, the notion of neogeography (Haklay 2013) may be of help here: a scholarly framework that promotes democratisation of technology use through the integration in technological design, development and use by ill-represented societal groups. Such a framework could underpin sponsored and government initiatives’ aims at assisting the empowerment of marginalised social fractions. Digital conservation also needs to develop frameworks for good practice and regulation (Maffey et al. 2015; Sandbrook 2015; Vas et al. 2015). The current absence of the latter may stimulate rapid growth of applications but potentially hamper the long-term sustainability of a budding field.

### Co-operation in conservation

The promotion of ‘digital justice’ and mitigation of skewed power relations through inclusion of a broad range of experts and stakeholders is all the more important when considering that in the non-profit sector, under which nature conservation practices tend to fall, innovation often builds on core technology developed elsewhere (e.g. military, large consumer markets) and is subsequently tailored to the needs of this ‘niche market’. It is argued by Joppa (2015) that nature conservation, on the whole, is ‘behind’ other domains (e.g. healthcare, education) in terms of digital innovation. While it could be asked whether this is the case on all fronts and whether it fundamentally matters (it may even have some advantages), it seems undeniable that “the current general approach is a patchwork of one-off projects and partnerships” (Joppa 2015). In a similar vein, co-operation between academia and the conservation community usually occurs through one-off programmes and there is much room for better interaction and more cooperation (Galán-Díaz et al. 2015). This seems to hold true both at the macro-level between large organisations, and at the smaller scale of individuals innovating to develop grass-root solutions to local problems.

### Interdisciplinary science and practice

Nature conservation has grown to become a diverse community of volunteers (naturalists and otherwise), biologists, ecologists, social scientists and policy-makers. It is recognised that the most productive co-operation emerges from interdisciplinary teams (Galán-Díaz et al. 2015; Jepson and Ladle 2015). The digitisation of nature conservation results in the expansion of that interdisciplinary community with computer scientists, engineers and programmers. While the demand for computer-savvy employees in nature conservation may indeed increase in years to come (Arts et al. 2013; Hampton et al. 2013), the well-known issues with interdisciplinary working will (again) have to be faced by conservationists adopting digital technology. Participants in interdisciplinary projects often lack the conceptual background to deal with different approaches from other disciplines (Pennington 2011). Different academic disciplines may differ in publication strategies (e.g. computer scientists favouring rapid publication in conference proceeding, ecologists preferring peer-reviewed journals). Ecology has been described as an individual-driven culture (Hampton et al. 2013) but many digital applications, especially involving big data, demand large-scale cooperation (Kelling et al. 2015). There is a potentially central role for social scientists in interdisciplinary digital innovation endeavours in nature conservation. As Adams (2009, p. xxxi) points out: “A social scientist on an interdisciplinary team in conservation is typically brought in late (…) has a lowly position and is asked (…) ‘what’s the answer to this question?’, when their training makes them want to ask ‘why is that the question you are asking?’”. The inclusion of researchers who focus on people and end-users from the outset will be likely to enhance the rate of learning. In this sense, a scientific discipline such as human–computer interaction seems to have much to offer to digital conservation. In any case, no simple solutions to interdisciplinary science and practice exist; it is essentially a social learning process (Pennington 2011). But if successful, inter- and cross-disciplinary partnerships can integrate methodologies and perspectives, possibly resulting in richer learning environments, the generation of deeper insight, more efficient working and higher impact, be it initially at a slower pace.

## Conclusion

Nature conservation is changing under the influence of digital technology. We have used the concept of digital conservation to describe this alteration and to consider its significance. On the basis of websites, scientific and grey literatures and other sources, we analysed the emerging field and distinguished five areas of application: data on nature, data on people, data integration and analysis, communication, and participatory governance (Fig. 1). Possibilities and problems were identified for each area—some of which already exist and others that are likely to happen in the future. Bearing in mind the growth of digital conservation, we warn against hypes, techno-fix thinking and unverified assumptions related to promise and short-term benefits. There is a strong need for the evaluation of impact and countering of the current bias towards good news narratives. We believe that a re-conceptualisation is desirable of technology as a dual-faced force that can be guided but not always controlled. Against a backdrop of increasingly converging technologies (Castells 2010), it may be more difficult to distinguish the digital from the non-digital in the future. This seems to hold true already for developments that potentially have a strong impact on nature conservation, such as synthetic biology (Kumar 2012; Redford et al. 2013b), DNA analysis of species and environmental traces (Larson 2007; Bohmann et al. 2014) and bio-robots (Wood et al. 2013). Hence, it is important to conceptualise digital conservation developments in a broad sense.

Nature conservation has a patchy record in terms of social impacts (e.g. the displacement of indigenous people from their land, fortress conservation, lack of stakeholder involvement in decision-making). Attention needs to be paid to who benefits (most) from digital conservation, and who does not (or who suffers from it); who is in control of information flows and processes; and how democratisation may be promoted. We note that there are opportunities for multi-sector co-operation—both on macro and micro levels—while ethical, good practice and assessment frameworks for (self-) regulation will need to be developed. We also argue that broad interdisciplinary science and academia-practice partnerships are central to a sustainable development of digital conservation.

Digital technology in nature conservation should be seen as something that is neither good nor bad. It is a force that will transform the work of conservation scientists, protected area managers and conservation organisations. Change will be driven partly through peer pressure, and partly through the inherent possibilities and problems that digital technology brings. We hope that more multi-sector, multi-discipline conferences and dialogues will follow to galvanise a digital conservation community of practice, research and policy. The concerted thinking and agenda-setting that should flow from such interactions will help to ensure that digital technology underpins key aims of nature conservation.

## Footnotes

1 Nature conservation: nature, natural, conservation, environment, biodiversity, ecosystem, ecology, flora, fauna, wildlife, wild, wilderness, natural area, national park, endangered species, community-based. Digital technology: digital, computer, smartphone, tablet, computational, technology, innovation, internet, web, online, ICT, electronic (e-), sensor, cyber, monitoring, database, network, software, hardware, support system, mobile, wireless.

2 Camera traps emerge as key tool in wildlife research (05–12–2011, Jeremy Hance). Yale Environment 360. http://e360.yale.edu/feature/camera_traps_emerge_as_key_tool_in_wildlife_research/2469/. Conservation Drones. Website: http://conservationdrones.org/. From cell phones to drones: How technology is helping conservation (28-04-2015, Enrique Gili). Deutsche Welle News. http://www.dw.de/global-ideas-technology-conservation-drones-software-fish-biodiversity/a-18412882/. How mobile technology is changing conservation (19-09-2014, Kathleen Garrigan). Blog, African Wildlife Foundation. http://www.awf.org/blog/how-mobile-technology-changing-conservation/. How technology is taking conservation science to the next level (02-04-2015, Karina Atkinson). The Guardian. http://www.theguardian.com/environment/2015/apr/02/how-technology-is-taking-conservation-science-to-the-next-level?utm_medium=twitter&utm_source=dlvr.it/. More than 300 sharks in Australia are now on Twitter (01-01-2014, Alan Yu). NPR news. http://www.npr.org/sections/alltechconsidered/2013/12/31/258670211/more-than-300-sharks-in-australia-are-now-on-twitter/. O'Reilly Media Animals. Website. http://animals.oreilly.com/. Rare dolphin sightings on the rise since release of dolphin-spotting app (20-02-2015, Olivia Wannan). Stuff news. http://www.stuff.co.nz/technology/apps/66427434/rare-dolphin-sightings-on-the-rise-since-release-of-dolphinspotting-app/. Sheep to warn of wolves via text message (06-08-2012) BBC News. http://www.bbc.co.uk/news/technology-19147403/. Technology for Nature. Website. http://www.technologyfornature.org/. Thermal imaging may save Hauraki Gulf whales (13-04-2015, Jack van Beynen). Stuff news. http://www.stuff.co.nz/environment/67712222/thermal-imaging-may-save-hauraki-gulf-whales/.

3 Tuna Fish Robot. Webpage, Robotics and Cybernetics Research Group Universidad, Politécnica de Madrid. http://www.robcib.etsii.upm.es/index.php/en/robots/.

4 Cyro. Webvideo, Virginia Tech: Autonomous Robotic Jellyfish. https://vimeo.com/62880818/.

5 BeeNav. Webpage, Rothamsted Research. http://www.rothamsted.ac.uk/news/beenav-bumblebees-cleverly-calculate-efficient-routes/.

6 Arnia Remote Bee Hive Monitoring. Webpage. http://www.arnia.co.uk/.

7 Blogging birds: The lives of red kites, told by computers. Website. http://redkite.abdn.ac.uk/.

8 5 ways regular people are tracking wildlife with personal tech (16-01-2015, Karen de Seve). National Geographic. http://news.nationalgeographic.com/news/2015/01/150114-crowdsourcing-science-animals-wildlife-conservation-technology-citizens/.

9 BirdTrack. App. http://www.bto.org/volunteer-surveys/birdtrack/about/. Conservation Hackathon—Tackling Conservation Challenges. Website. http://conservationhackathon.org/. Naturelocator. Website. http://naturelocator.org/. Zooniverse. Website. https://www.zooniverse.org/.

10 iBats: Indicator Bats Program. Website. http://www.ibats.org.uk/. KeyToNature: A new e-way to discover biodiversity. Website. http://www.keytonature.eu/. Leafsnap: An Electronic Field Guide. Website. http://leafsnap.com/. New Forest Cicada Project. App. http://newforestcicada.info/app/.

11 Notes from Nature. Website. http://www.notesfromnature.org/.

12 Greendex 2014: Consumer Choice and the Environment—A worldwide Tracking Survey. http://environment.nationalgeographic.com/environment/greendex/.

13 Wildlife Crime Tech Challenge. Website: http://wildlifecrimetech.org/index/. Drones join war on rhino poachers in South Africa (27-05-2013, Aislinn Laing). The Telegraph. http://www.telegraph.co.uk/news/worldnews/africaandindianocean/southafrica/10082727/Drones-join-war-on-rhino-poachers-in-South-Africa.html/. How killing elephants finances terror in Africa (12–08–2015, Bryan Christy). National Geographic. http://www.nationalgeographic.com/tracking-ivory/article.html/. Kenya to microchip every rhino in anti-poaching drive (16–10–2013). BBC News. http://www.bbc.com/news/world-africa-24558136/. Rhino horn camera ‘could save rhinos from extinction’ (20–07–2015). BBC News. http://www.bbc.com/news/science-environment-33590436/. Topher White: What can save the rainforest? Your used cell phone (03-03-2015, Topher White). TED presentation. https://www.youtube.com/watch?v=xPK2Ch90xWo/.

14 Brazilian police and scientists team up to crack down on illegal timber trade (08-07-2015, Jonathan Mason). World Resources Institute. http://www.wri.org/blog/2015/07/brazilian-police-and-scientists-team-crack-down-illegal-timber-trade/.

15 Forbidden Data: Wyoming just criminalised citizen science (11-05-2015, Justin Pidot). Slate News. http://www.slate.com/articles/health_and_science/science/2015/05/wyoming_law_against_data_collection_protecting_ranchers_by_ignoring_the.html?wpsrc=sh_all_dt_tw_top/.

16 Global Biodiversity Information Facility (GBIF): Free and Open Access to Biodiversity Data. Website. http://www.gbif.org/.

17 National Biodiversity Network (NBN) Gateway. Website. http://www.nbn.org.uk/.

18 Darwin Core Project. Website. http://rs.tdwg.org/dwc/.

19 Speciesbank.com. Website. http://www.speciesbanking.com/.

20 Can you use big data to track an elephant poacher? (12–06–2015, Kalev Leetaru). Foreign Policy—Voice. http://foreignpolicy.com/2015/06/12/can-you-use-big-data-to-track-an-elephant-poacher/. TrackLab for wildlife tracking. Software. http://www.noldus.com/innovationworks/products/tracklab/wildlife.

21 In some cases internet-supported social media and platforms help to connect animals with animals (Apps for apes, Orangutan Outreach. Website. http://redapes.org/multimedia/apps-for-apes/) or 'enable' humans to write to trees (When you give a tree an email address, 10–07–2015, Adrienne LaFrance—Citylab. http://www.citylab.com/tech/2015/07/when-you-give-a-tree-an-email-address/398219/).

22 Open Air Laboratories (OPAL). Website. http://www.opalexplorenature.org/.

23 eBird. Website. http://ebird.org/.

24 iNaturalist. Website. http://www.inaturalist.org/.

25 Atlas of Living Australia. Website. http://www.ala.org.au/.

26 WikiAves. Website. http://www.wikiaves.com/.

27 Push to revise conservation law as Indonesians post wildlife crimes to Facebook (10–07–2015, Fidelis Satriastanti). Mongabay. http://news.mongabay.com/2015/07/push-to-revise-conservation-law-as-indonesians-post-wildlife-crimes-to-facebook/.

28 Eggcellent citizen science: evolution of camouflage in bird eggs (27–08–2014, GrrlScientist). The Guardian, Science. http://www.theguardian.com/science/grrlscientist/2014/aug/27/eggcellent-citizen-science-evolution-of-camouflage-in-bird-eggs/. 10 Environmental games that teach kids about earth, ecology, and conservation (03–09–2010, Saikat Basu). Makeuseof. http://www.makeuseof.com/tag/10-environmental-games-teach-kids-earth-ecology-conservation/. Gamification in sustainable development (14–03–2014, Erik Swan). Newsletter, BEAHRS, Environmental Leadership Program, UC Berkeley. http://beahrselp.berkeley.edu/newsletter/gamification-in-sustainable-development/. We are the rangers. United for Wildlife. Game. http://wearetherangers.com/.

29 Wildtime. App. http://wildtime.projectwildthing.com/.

30 National Geographic—Interactive Experience: Son Doong in 360°—Dive into ‘infinity’ with dizzying views of a colossal cave (20–05–2015, Jane Lee and Martin Edström). Interactive website. http://news.nationalgeographic.com/2015/05/150520-infinity-cave-son-doong-vietnam-virtual-tour-photography-conservation/ Nautilus Live—Explore the ocean LIVE with Dr. Robert Ballard and the Corps of Exploration. Website. http://www.nautiluslive.org/ Opti-hunting (25–10–2012, Paul Jepson). http://www.geog.ox.ac.uk/staff/pjepson-opti-hunting%20proposal_25Oct12.pdf/ Reality is too confining (24–10–2014, Amy Westervelt). Conservation Magazine, University of Washington. http://conservationmagazine.org/2014/10/reality-is-too-confining/. Tele Echo Tube. Website. http://hhkobayashi.com/tele-echo-tube/.

31 Ecosia. Internet search engine: https://www.ecosia.org/.

32 Map-Me. Webdocument. http://map-me.org/.

33 Extreme Citizen Science group, University College London. Website: http://www.ucl.ac.uk/excites/.

34 Mapping for Rights. Website. http://www.mappingforrights.org/.
